# Childhood Immunization in Ethiopia: Accuracy of Maternal Recall Compared to Vaccination Cards

**DOI:** 10.3390/vaccines7020048

**Published:** 2019-06-07

**Authors:** Julia M. Porth, Abram L. Wagner, Yemesrach A. Tefera, Matthew L. Boulton

**Affiliations:** 1 Department of Epidemiology, School of Public Health, University of Michigan, Ann Arbor, MI 48109, USA; awag@umich.edu (A.L.W.); mboulton@umich.edu (M.L.B.); 2 Department of Public Health, St. Paul’s Hospital Millennium Medical College, Addis Ababa 1271, Ethiopia; yemieye197@gmail.com; 3 Department of Internal Medicine, Division of Infectious Diseases, University of Michigan Medical School, Ann Arbor, MI 48109, USA

**Keywords:** childhood immunization, maternal recall, caregivers, sensitivity and specificity, accuracy, Ethiopia

## Abstract

Health surveys conducted in low- and middle-income countries typically estimate childhood vaccination status based on written vaccination cards, maternal recall (when cards are not available), or a combination of both. This analysis aimed to assess the accuracy of maternal recall of a child’s vaccination status in Ethiopia. Data came from a 2016 cross-sectional study conducted in the Southern Nations, Nationalities, and Peoples’ (SNNP) Region of Ethiopia. Vaccine doses received by a given 12–23-month-old child were recorded from both a vaccination card and based on maternal recall and then compared. Concordance, sensitivity, specificity, positive predictive value (PPV), negative predictive value (NPV), and Cohen’s Kappa were calculated. Estimates of full immunization coverage were similar when collected via vaccination card (75%) and maternal recall (74%). For fully vaccinated children, comparison of maternal recall versus vaccination card showed high concordance (96%), sensitivity (97%), specificity (93%), PPV (98%), NPV (92%), and Kappa (90%). Accuracy of maternal recall of a child’s vaccination status is high in the SNNP region of Ethiopia. Although determination of vaccination status via vaccination card is preferred since it constitutes a written record, maternal recall can also be used with confidence when vaccination cards are not available.

## 1. Introduction

Since its inception in the 1970s, the World Health Organization’s Expanded Program on Immunization (EPI) has aimed to increase childhood vaccination coverage globally by making recommendations for childhood vaccination schedules and working with ministries of health to provide those vaccines, for free, to children [1]. Public health programming for these childhood vaccination services relies on valid estimates of children’s vaccination status in order to develop accurate estimates of population-level coverage. Health surveys conducted in low- and middle-income countries often assess vaccination status using maternal (caregiver) recall when vaccination cards are unavailable and, as such, it is important to understand the validity of caregiver recall compared to a print vaccination card in documenting a child’s immunization status. 

Prior studies have reported mixed findings regarding the accuracy of caregiver recall of child vaccination status. Reports from Tanzania [2], Sudan [3], India [4], Egypt [5], Bangladesh [6], and Canada [7] all reported high accuracy and/or validity of parental recall of child vaccination status. However, a 2018 systematic review of the quality of caregiver recall in low- and middle-income countries [8] found highly variable results. Studies from the United States [9,10,11,12] and India [13] show poor accuracy and validity of parental recall, findings that have been supported by a global systematic review [14]. Further, it has been shown that accuracy and validity of recall may vary by specific vaccine and vaccine dose administered. A study from Tanzania found that while recall sensitivity and concordance were lower for the measles vaccine than other vaccines in the childhood series, specificity, unadjusted Kappa, and both over and under-reporting of vaccine receipt were substantially higher for the measles vaccine than other vaccines in the series. Further, recall of the Bacillus Calmette–Guérin (BCG) vaccine birth dose demonstrated the highest concordance and prevalence-adjusted bias-adjusted Kappa (PABAK) of all vaccines in the schedule [2]. Subsequently, recommendations for relying on use of caregiver recall vary depending on the context, with many studies advising against its use to assess the vaccination status of individual children [7,9,10,11,12,13,15,16,17] but endorsing it for purposes of vaccination coverage assessments [2,3,4,5,6,8,18]. 

Developing correct estimates of vaccination coverage to inform immunization strategy development is particularly important in the sub-Saharan African region, as many countries in the region are at high risk for vaccine-preventable disease outbreaks. The proportion of vaccine-preventable disease deaths is higher in sub-Saharan Africa than in other regions than the world [19] and vaccination coverage is low in many of the region’s countries, indicating the need for continued efforts to improve vaccination uptake in sub-Saharan Africa. In fact, of the approximately 20 million children globally who did not receive all three recommended doses of the diphtheria, tetanus, and pertussis (DTP) vaccine in 2017, 60% of those children are from only 10 countries; 5 of those 10 countries are in sub-Saharan Africa (i.e., Angola, Democratic Republic of the Congo, Ethiopia, Nigeria, and South Africa) [20]. Another commonality between these five countries, and many other sub-Saharan African countries, is the dependence on parental recall of child’s vaccination status. Retention of vaccination cards for children aged 12–23 months ranges from 26% in the Democratic Republic of the Congo [21] to 66% in South Africa [22]. This high reliance on parental recall demonstrates the need to understand if recall accurately represents a child’s vaccination status with comparable information yield relative to vaccination cards.

With a population of approximately 108 million, Ethiopia is the second most populous country in sub-Saharan Africa [23]. It has one of the most complex vaccination schedules in the region, with six vaccines provided for free to all children through the EPI: the BCG vaccine (for tuberculosis), the oral polio vaccine (OPV), the pentavalent vaccine (includes DTP, hepatitis B (HBV), and *Haemophilus influenzae* type b (Hib)), the pneumococcal conjugate vaccine (PCV), the rotavirus vaccine, and the measles-containing vaccine [24,25]. With only 33% of children aged 12–23 months fully vaccinated [25], Ethiopia is far below the World Health Organization target of 90% coverage, and, as previously noted, is among the 10 countries globally with the highest nonvaccination with DTP3 [20]. It is in this context that vaccine-preventable morbidity and mortality remain high, with diarrheal diseases, respiratory infections, and tuberculosis among the top five causes of death for children under five [26]. 

The majority of existing studies on recall have been conducted in high-income countries, including the United States [9,10,11,12,17] Canada [7], and the United Kingdom [15] As the context of healthcare and vaccination in high-income countries are quite different than in low- and middle-income countries, the degree to which these studies are applicable to Ethiopia and other African nations is not clear. Although there have been studies of caregiver recall accuracy in some low- and middle-income countries [2,3,4,5,6,13,16], no studies have examined the accuracy of parental recall and its predictors in Ethiopia. This analysis aimed to assess the validity of maternal recall of a child’s vaccination status in southern Ethiopia. Given the large population size, complex EPI schedule, low vaccination coverage, and high burden of vaccine-preventable diseases, Ethiopia is an ideal location to study parental recall of child vaccination; understanding the accuracy of parental recall in Ethiopia may provide the ability to inform efforts to assess and improve vaccination coverage in other low-income countries, particularly those on the African continent. 

## 2. Materials and Methods 

Data for this analysis came from a cross-sectional survey conducted in the summer of 2016 in Worabe town in the Southern Nations, Nationalities, and Peoples’ Region of Ethiopia. The 2007 Ethiopian census reported 27,852 residents in Worabe town; 15% of these residents were under five years of age [27]. Details of the survey methodology are published elsewhere [27]. Briefly, community health workers traveled to each of the two *kebeles* (equivalent to neighborhoods) in Worabe town and determined a random starting point using a random number chart. From this starting point, all households in each *kebele* were enumerated. Interviewers then started at the random starting point and visited each home to see if at least one child aged 12–23 months lived there and interviewed the children’s mother. Subsequent neighboring households were visited until the sample quota of 270 homes per *kebele* was obtained. Data collected included sociodemographic characteristics, health services utilization, child characteristics, child vaccination status, and maternal knowledge regarding vaccines and vaccine-preventable diseases. Information about the child’s receipt of individual vaccines and number of doses was obtained from both maternal recall and vaccination cards, if available [27].

Study participants were initially asked to recall their child’s vaccination status (i.e., maternal recall) and then to provide the child’s vaccination card; only those participants who did both were included in the study. Several variables were created for this analysis. When the vaccination card indicated that a child received a specific dose but no date was recorded, the child was considered vaccinated. For the oral polio vaccine, the pentavalent vaccine, the pneumococcal conjugate vaccine, and the rotavirus vaccine, a new variable was generated that summed the number of doses the child received of each vaccine, according to the vaccination card. A binary variable was used to indicate whether the child was fully immunized; a child was considered fully vaccinated if he or she received one dose of BCG, three doses of OPV, three doses of the pentavalent vaccine, three doses of PCV, two doses of the rotavirus vaccine, and one dose of the measles-containing vaccine. Child’s vaccination status according to maternal recall was treated similarly. Variables were crafted to indicate whether vaccination status via recall and card were the same in children who were fully vaccinated and for each vaccine. Some categories of sociodemographic variables (religion, occupation, parity, marital status, etc.) were collapsed to allow sufficient cell sizes to conduct the appropriate analyses. 

Vaccination coverage was calculated by source (i.e., recall vs. card) and compared. Net reporting bias was defined as the difference between vaccination coverage obtained via recall versus vaccination card. To determine the validity of maternal recall in correctly identifying a child’s vaccination status, concordance, sensitivity, specificity, positive predictive value, negative predictive value, and Kappa were computed using vaccination status obtained from the vaccination card as the gold standard. Accuracy of maternal recall was assessed as the percent of mothers who correctly recalled their child’s vaccination status (compared to the vaccination card). False positives were the percent of mothers who reported their child received a vaccine when the card indicated the child had not received that vaccine (i.e., mothers who overestimated their child’s vaccination status, Table 1). False negatives were the percent of mothers who reported their child did not receive a vaccine when the vaccination card designated the child had received the vaccine (i.e., mothers who underestimated their child’s vaccination status). 

All analyses were conducted for full vaccination and each vaccine type. Due to small sample sizes, for vaccines provided in a series of multiple doses (i.e., the pentavalent vaccine), analyses only compared whether maternal recall and the vaccination card reported receipt of any doses of the vaccine rather than maternal recall of specific doses of the vaccine. Associations between maternal recall accuracy and demographic factors were examined using a chi-square analysis. Analyses were conducted using SAS version 9.4 (SAS Institute, Cary, NC, USA).

Approval for data collection was granted by the Institutional Review Board at St. Paul’s Hospital Millennium Medical College and the Worabe Health Bureau approved study activities in their region. Verbal informed consent was attained by data collectors prior to participant enrollment. This analysis was approved by the University of Michigan Health Sciences and Behavioral Sciences Institutional Review Board.

## 3. Results

Interviewers contacted 540 mothers and 56 declined to participate, leaving a total of 484 (90%) mothers in the original survey. Of these, 247 participants were excluded from this analysis because they did not have their child’s vaccination card and 5 individuals were eliminated because their child’s age was outside of the 12–23 months age range. These exclusions resulted in a final sample size of 232 individuals for this analysis. By design, only mothers were interviewed (i.e., no other family members were interviewed). Mothers’ mean age was 28 years and the majority of women were married (97%), Muslim (87%), and worked as homemakers (60%, Table 2). Half of the children were females (53%) and child’s mean age was 17 months. 

Estimates of full vaccination coverage were high based on both vaccination cards (75% fully vaccinated) and maternal recall (74%, Table 3). Net reporting bias was negative for estimates of full vaccination and receipt of all recommended OPV doses, indicating estimates of vaccination status from cards were higher than from maternal recall. Conversely, net reporting bias was positive for receipt of BCG vaccine, all recommended pentavalent vaccine doses, all recommended PCV doses, both recommended rotavirus doses, and measles vaccine, which suggests mothers overestimated receipt of individual vaccines, though the magnitudes of these measures were small (Table 3). Most vaccinated children received the recommended number of doses of each vaccine: one dose of the BCG vaccine, three (or four) doses of the OPV vaccine, three doses of the PCV vaccine, and two doses of the rotavirus vaccine (Table 4).

Concordance between maternal recall and vaccination card was high, with all measures over 95%. Kappa, however, was quite variable; Kappa values were high for full vaccination and receipt of the measles vaccine but modest for receipt of all other vaccines (Table 5). Maternal recall was highly sensitive with measures for all vaccines over 97%, indicating that of the children whose vaccination cards showed they had received a vaccine, over 97% of mothers correctly identified their child as vaccinated with that vaccine. Specificity of maternal recall was high for full vaccination (93%) and the measles vaccine (92%) but generally low for receipt of all other vaccines. This suggests that of the children whose vaccination cards showed they had not received a vaccine, few mothers correctly identified their child as unvaccinated. Similar to sensitivity, positive predictive values were over 97% for all vaccines; of children whose mothers indicated they were vaccinated, over 97% were truly vaccinated according to their vaccination card. Negative predictive value was also generally high, indicating that of children whose mothers indicated that they were not vaccinated, most were not vaccinated based on the card.

Recall accuracy was extremely high for full vaccination (96%) and receipt of each type of vaccine (Table 6). False positive and false negative reports of vaccine receipt were uncommon for full vaccination (2% of reports were false positives and 2% were false negatives) and each vaccine. No statistically significant associations were found between recall accuracy and demographic factors (Table 7).

## 4. Discussion

This analysis examined the accuracy of maternal recall of child vaccination status in a southern region of Ethiopia. We found generally high levels of concordance between maternal recall and vaccination card data. While mothers were more likely to overestimate the number of vaccine doses their child had received, they paradoxically tended to slightly underestimate whether their child was fully vaccinated with all recommended vaccine doses. A recent study from Tanzania that compared vaccination status via vaccination card and maternal recall similarly found mothers tended to overestimate receipt of individual doses of vaccine [2]. It has been hypothesized that overestimation in these circumstances may be due to social desirability bias; that is, women may feel that having a vaccinated child is more favorably perceived and may therefore report receipt of vaccine doses irrespective of their child’s actual vaccination status [2]. Given that female community health workers were collecting data from mothers in our study, it may be that the sense of social pressure to report children as vaccinated was particularly acute. Conversely, it is also possible that the mothers’ recall was accurate rather than representing an overestimation if a child received vaccinations that were not recorded on the vaccination card. This scenario might be more likely, for example, if a caregiver forgets to bring the vaccination card when their child is vaccinated, if perhaps in situations where the child is vaccinated at an appointment when the primary purpose of the visit was for something other than vaccination, or if the child was immunized as part of a special immunization activity (e.g., catch-up campaign or national immunization day), which may reduce the probability that vaccination(s) will be appropriately recorded on the card. In fact, when asked if their child had received vaccines other than those listed on the card, 99% of the women in our study responded affirmatively. Other studies have reported limitations of vaccination-card-reported data. One study from India that examined prospectively obtained vaccination information compared to vaccination cards and retrospective recall of vaccination status found that the vaccination cards of 70% of the sampled children were incompletely and/or inaccurately filled out [13]. Another study from Egypt noted problems with transcription of correct information onto the vaccination cards and suggested that some inconsistencies in maternal recall accuracy were due to vaccination card errors rather than faulty maternal recall [5].

Sensitivity of maternal recall was extremely high in this study, above 97% for full vaccination and also for receipt of specific vaccines. However, specificity was more variable, at over 90% for full vaccination status and for receipt of the measles vaccine, but much lower (25–50%) for receipt of BCG, pentavalent, PCV, and rotavirus vaccines. Low specificity is especially concerning in this context as it potentially represents a worst-case scenario—a mother who thinks her child is vaccinated with a specific vaccine (and therefore less likely to vaccinate with that same vaccine in the future) when the child is actually unvaccinated and therefore unprotected against disease. These findings align with low recall specificity of BCG, OPV, and DPT which was found in a prior Tanzanian study of maternal recall [2]. It may be that women conceptualize their child’s vaccination status in terms of whether future visits to the health clinic will be needed or are necessary. Full vaccination of their child(ren) is a commonly articulated goal by women and, as such, it may be they are well informed about whether their child has received all necessary vaccines (and therefore do not need to return for future vaccinations) but may not know which specific vaccines are included in the series nor which her child has received. It is also likely that social desirability bias may play a role here, too. A woman may feel more comfortable telling a health worker that her child is not fully vaccinated (potentially interpretable as vaccination delay but with intentions to complete) as opposed to informing a health worker about the specific recommended vaccines and/or doses that her child has not yet received. 

### Strengths and Limitations

Like all studies, this one has limitations. The small sample size was a barrier to conducting analyses with sufficient power to support conclusions and the findings should be interpreted in that context. This may explain why statistically significant associations between sociodemographic factors and accuracy of maternal recall that have been identified in other studies (e.g., caregiver age and education [2]) were not significant in this analysis. Vaccines with multiple doses in a series only compared whether maternal recall and the vaccination card reported receipt of any doses of the vaccine, which limits conclusions regarding a mother’s ability to correctly identify the number of doses her child received. Due to the small number of women who inaccurately identified their child’s vaccination status, we were unable to run more nuanced analyses examining validity of recall of particular numbers of doses of vaccine. Women who could not produce a vaccination card for their child (51% of the initial sample) were excluded from this study. Vaccination card retention is related to a child’s immunization status and may demonstrate a stronger maternal interest in vaccination services and thus potentially introducing selection bias in the study population [28]. Worabe, the site of this study, is a predominantly Muslim town—whereas the majority population in Ethiopia is Ethiopian Orthodox. Previous studies have found religion to be associated with both full vaccination [29] and vaccine nonreceipt [30] in Ethiopia. A larger, nationally representative survey comparing vaccination status via maternal recall and vaccination card would improve upon the sample size and the limits to generalizability present in this study. Additionally, studies have shown health provider records of vaccination receipt are generally more accurate than vaccination cards [14]. A comparison of maternal recall to health clinic records (instead of to vaccination cards) may provide a better estimate of the accuracy of maternal recall. However, Worabe health clinic records were not available to the study team. This study also has the important strength of being the first to examine the validity of maternal recall of a child’s vaccination status in Ethiopia. Also, in addition to examining validity of maternal recall of full vaccination, this study examined the validity of maternal recall of each vaccine included in the Expanded Program on Immunization series. 

## 5. Conclusions

Given that many health surveys rely on both vaccination cards and maternal recall to estimate childhood vaccination coverage and maternal recall validity, it is important to understand if maternal recall is a valid reflection of true vaccination status. The results of this study suggest that the accuracy of maternal recall of a child’s vaccination status was generally high. Although determination of vaccination status via vaccination card is preferred, maternal recall should be used when cards are not available. The results of this and future related studies could help researchers and public health officials correctly estimate vaccination coverage in Ethiopia and elsewhere, which is necessary for appropriate planning of vaccination programs and outreach.

## Figures and Tables

**Table 1:** 2 × 2 table used for accuracy calculations.

Agreement of Card and Recall		Vaccination Status via Card		
		Yes	No	
Vaccination status via recall	Yes	True positives (TP)	False positives (FP)	TP + FP
	No	False negatives (FN)	True negatives (TN)	FN + TN
		TP + FN	FP + TN	Total

**Table 2:** Descriptive statistics of sample of women and children included in accuracy analysis (n = 232).

Variable	Frequency (Mean)	Percent (Standard Deviation)
Child characteristics		
Child’s sex		
Female	122	52.59
Male	110	47.41
Child’s age		
11–15 months	82	35.34
16–20 months	76	32.76
Older than 20 months	74	31.90
Child’s age, continuous	(17.72)	(3.93)
Child’s birth order		
1	76	32.76
2	53	22.84
3	42	18.10
4 or more	61	26.29
Maternal characteristics		
Mother’s age, continuous	(27.81)	(5.01)
Mother’s age, categorical		
25 or younger	75	32.33
25–29	66	28.45
Older than 29	62	26.72
Do not know or missing	29	12.50
Family monthly income, continuous	(1993.97)	(1436.11)
Family monthly income, categorical		
Less than or equal to 1100 birr	55	23.71
Between 1100 and 2000 birr	52	22.41
Greater than 2000 birr	54	23.28
Missing	71	30.60
Religion		
Muslim	201	86.64
Orthodox	19	8.19
Catholic or Protestant	12	5.17
Religion, collapsed		
Muslim	201	86.64
Other	31	13.36
Marital Status		
Married	226	97.41
Other	6	2.59
Number of children mother has		
1	74	31.90
2	54	23.28
3	39	16.81
4 or more	65	28.02
Occupation of mother		
Housewife	139	60.17
Merchant	33	14.29
Government or private institution worker	30	12.99
Farmer	16	6.93
Daily job	8	3.46
Student	5	2.16
Occupation of mother, collapsed		
Housewife	139	60.17
Other, employed outside of the home	92	39.83
Education level of mother		
No formal education	78	33.91
Elementary	89	38.70
High school or higher	63	27.39
How long does it take to reach the nearest immunization		
center (in minutes)?		
30 min or less	107	46.72
More than 30 min	122	53.28
Have you ever taken your child to a health facility to get a health service other than vaccination?		
Yes	226	97.41
No	6	2.59

**Table 3:** Vaccination coverage via recall and card and net reporting bias.

Vaccine	Coverage via Recall (%)	Coverage via Card (%)	Net Reporting Bias
Full vaccination	74.14	74.57	−0.43
BCG	98.70	97.84	0.86
OPV ^a^	94.37	94.8	−0.43
Pentavalent ^b^	86.58	84.05	2.53
PCV ^b^	85.78	83.62	2.16
Rotavirus ^c^	94.40	93.10	1.30
Measles	84.05	83.62	0.43

^a^ Coverage indicates receipt of either three or four doses of OPV vaccine. ^b^ Coverage indicates receipt of all three recommended doses of the vaccine. ^c^ Coverage indicates receipt of both of the two recommended doses of rotavirus vaccine. BCG—Bacillus Calmette–Guérin; OPV—oral polio vaccine; PCV—pneumococcal conjugate vaccine.

**Table 4:** Vaccination status of children, according to vaccination card and maternal recall.

	Vaccination Card		Maternal Recall	
Vaccination Status, as per Vaccination Card	Frequency	Percent	Frequency	Percent
Receipt of BCG vaccine	226	97.84	227	98.70
Number of doses of OPV child has received				
0	2	0.87	2	0.87
1	2	0.87	5	2.16
2	8	3.46	6	2.60
3	28	12.12	40	17.32
4	191	82.68	178	77.06
Number of doses of pentavalent vaccine child has received				
0	4	1.72	1	0.43
1	8	3.45	8	3.46
2	25	10.78	22	9.52
3	195	84.05	200	86.58
Number of doses of PCV vaccine child has received				
0	4	1.72	2	0.86
1	8	3.45	8	3.45
2	26	11.21	23	9.91
3	194	83.62	199	85.78
Number of doses of rotavirus vaccine child has received				
0	5	2.16	2	0.86
1	11	4.74	9	3.88
2	216	93.10	219	94.40
3	0	0.00	2	0.86
Child has received measles vaccine	194	83.62	195	84.05
Child is fully vaccinated	173	74.57	172	74.14

**Table 5:** Measures of recall accuracy.

Measure of Accuracy	Full Vaccination	BCG	OPV ^a,b^	Pentavalent ^a,b^	PCV ^a,b^	Rotavirus ^a,b^	Measles
Concordance, % (95% CI)	96.12 (92.76, 98.21)	98.69 (96.22, 99.73)	98.28 (95.64, 99.53)	98.71 (96.27, 99.73)	99.14 (96.92, 99.90)	98.71 (96.27, 99.73)	97.84 (95.04, 99.30)
Cohen’s Kappa Coefficient, % (95% CI)	89.83 (83.32, 96.33)	56.49 (12.24, 100.00)	— ^c^	39.58 (−14.54, 93.71)	66.28 (22.47, 100.00)	56.61 (12.66, 100.00)	92.05 (85.16, 98.93)
Sensitivity, % (95% CI)	97.11 (93.38, 99.06)	99.56 (97.55, 99.99)	99.13 (96.89, 99.89)	100.00 (98.40, 1.00.00)	100 (98.40, 100.00)	100.0 (98.39, 100.00)	98.97 (96.33, 99.87)
Specificity, % (95% CI)	93.22 (83.54, 98.12)	50.00 (6.76, 93.24)	— ^c^	25.00 (0.63, 80.59)	50.00 (6.67, 93.24)	40.00 (5.27, 85.34)	92.11 (78.62, 98.34)
Positive Predictive Value, % (95% CI)	97.67 (94.15, 99.36)	99.12 (96.84, 99.89)	99.13 (96.89, 99.89)	98.70 (96.25, 99.73)	99.13 (96.89, 99.89)	98.70 (96.24, 99.73)	98.46 (95.57, 99.68)
Negative Predictive Value, % (95% CI)	91.67 (81.61, 97.24)	66.67 (9.43, 99.16)	— ^c^	100.00 (2.50, 100.00)	100.00 (15.81, 100.00)	100.00 (15.81, 100.00)	94.59 (81.81, 99.34)

^a^ Measures of validity for this vaccine compare whether the child’s vaccination card and maternal recall indicate receipt of at least one dose in the series. ^b^ Contains a zero cell count. ^c^ Cannot calculate due to zero cell.

**Table 6:** Accuracy, recall false positives, and recall false negatives by vaccine.

Accuracy of Recall	Frequency	Percent
Accuracy of caregiver recall compared to vaccination card—full vaccination		
Accurate (card and recall have the same value)	223	96.12
False positive (card says child is not vaccinated but recall says child is vaccinated) ^a^	4	1.72
False negative (card says child is vaccinated but recall says child is not vaccinated) ^b^	5	2.16
False positive or false negative (combined)	9	3.88
Accuracy of caregiver recall compared to vaccination card—BCG		
Accurate (card and recall have the same value)	226	98.69
False positive (card says child is not vaccinated but recall says child is vaccinated) ^a^	2	0.87
False negative (card says child is vaccinated but recall says child is not vaccinated) ^b^	1	0.44
False positive or false negative (combined)	3	1.31
Accuracy of caregiver recall compared to vaccination card—Pentavalent		
Accurate (card and recall have the same value)	229	98.71
False positive (card says child is not vaccinated but recall says child is vaccinated) ^a^	3	1.29
False negative (card says child is vaccinated but recall says child is not vaccinated) ^b^	0	0.00
False positive or false negative (combined)	3	1.29
Accuracy of caregiver recall compared to vaccination card—Rotavirus		
Accurate (card and recall have the same value)	229	98.71
False positive (card says child is not vaccinated but recall says child is vaccinated) ^a^	3	1.29
False negative (card says child is vaccinated but recall says child is not vaccinated) ^b^	0	0.00
False positive or false negative (combined)	3	1.29
Accuracy of caregiver recall compared to vaccination card—Measles		
Accurate (card and recall have the same value)	227	97.84
False positive (card says child is not vaccinated but recall says child is vaccinated) ^a^	3	1.29
False negative (card says child is vaccinated but recall says child is not vaccinated) ^b^	2	0.86
False positive or false negative (combined)	5	2.16

^a^ Overestimation of vaccination status. ^b^ Underestimation of vaccination status.

**Table 7:** Chi-square statistics of sociodemographic variables and whether the maternal recall demonstrates accurate vaccination status or false positive or false negative.

Variable	Chi-Square Test Statistic	*p*-Value	Fisher’s Exact Test Two-Sided Probability	% of Cells with Expected Counts <5
Child sex	0.0331	0.8556	1.0000	50
Child age	0.8526	0.6529	0.6388	50
Birth order	1.9914	0.5742	0.6592	50
Caregiver age	5.0750	0.1664	0.2349	50
Education	1.1569	0.5608	0.6899	50
Religion	3.2259	0.0725	0.1038	25
Marital status	0.2486	0.6181	1.0000	25
Parity	1.6841	0.6405	0.7112	50
Occupation	0.9667	0.3255	0.4893	25
Family monthly income	3.0426	0.3851	0.4803	50
Distance to vaccination site	0.0196	0.8887	1.0000	50
Ever been to a health facility for a health service other than vaccination	0.2486	0.6181	1.0000	25
